# Association between Daily Hospital Outpatient Visits for Accidents and Daily Ambient Air Temperatures in an Industrial City

**DOI:** 10.1371/journal.pone.0145003

**Published:** 2016-01-27

**Authors:** Tang-Tat Chau, Kuo-Ying Wang

**Affiliations:** 1 Department of Family Medicine, Taiwan Landseed Hospital, Tao-Yuan, Taiwan; 2 Department of Atmospheric Sciences, National Central University, Chung-Li, Taiwan; Tsinghua University, CHINA

## Abstract

An accident is an unwanted hazard to a person. However, accidents occur. In this work, we search for correlations between daily accident rates and environmental factors. To study daily hospital outpatients who were admitted for accidents during a 5-year period, 2007–2011, we analyzed data regarding 168,366 outpatients using univariate regression models; we also used multivariable regression models to account for confounding factors. Our analysis indicates that the number of male outpatients admitted for accidents was approximately 1.31 to 1.47 times the number of female outpatients (*P* < 0.0001). Of the 12 parameters (regarding air pollution and meteorology) considered, only daily temperature exhibited consistent and significant correlations with the daily number of hospital outpatient visits for accidents throughout the 5-year analysis period. The univariate regression models indicate that older people (greater than 66 years old) had the fewest accidents per 1-degree increase in temperature, followed by young people (0–15 years old). Middle-aged people (16–65 years old) were the group of outpatients that were more prone to accidents, with an increase in accident rates of 0.8–1.2 accidents per degree increase in temperature. The multivariable regression models also reveal that the temperature variation was the dominant factor in determining the daily number of outpatient visits for accidents. Our further multivariable model analysis of temperature with respect to air pollution variables show that, through the increases in emissions and concentrations of CO, photochemical O3 production and NO2 loss in the ambient air, increases in vehicular emissions are associated with increases in temperatures. As such, increases in hospital visits for accidents are related to vehicular emissions and usage. This finding is consistent with clinical experience which shows about 60% to 80% of accidents are related to traffic, followed by accidents occurred in work place.

## Introduction

Safety is a top priority when a person participates in any form of daily activity. We are fortunate if we are safe every day. However, accidents do occur. Is there a background trend that reveals a hidden fact about all accidents that occur? Ambient temperature (heat) has been demonstrated to be related to aggression, with an increase in temperature leading to an increased occurrence of human violence [1]. In professional baseball, a positive and significant relationship between temperature and the number of batters hit by pitches per game has been observed; this result suggests that higher temperatures cause pitchers to become more aggressive when pitching to batters [2]. A direct linear increase in horn honking with increasing temperature has also been observed [3]. However, a study of accident risk in a Swedish town concluded that high temperature (and rain) do not increase the risk of accidents for low-speed buses [4].

In this work, we used the daily number of hospital outpatient visits for accidents and daily temperature data to study the correlation between the occurrence of accidents and temperature in an industrial area in northern Taiwan during the period of 2007–2011. The use of daily hospital medical records ensures better data quality over a long period [5]. This long studying period enabled us to obtain and test the statistical significance of our results. In the regression model analysis presented below, we also included and analyzed correlations between the number of hospital visits for accidents and other environmental factors (meteorological factors in addition to temperature and air pollution factors, which have been demonstrated to have health effects).

## Materials and Methods

### Ethics Statement

The statistics regarding the daily number of hospital visits used in this work are openly published each year by the hospital [6–9]. Only the frequencies of visits for diseases were used in the study. No details of patient’s personal information were involved in this work. Consequently, neither ethics committee approval nor written consent was required.

### Hospital Outpatient Visit Data

**Fig 1:**
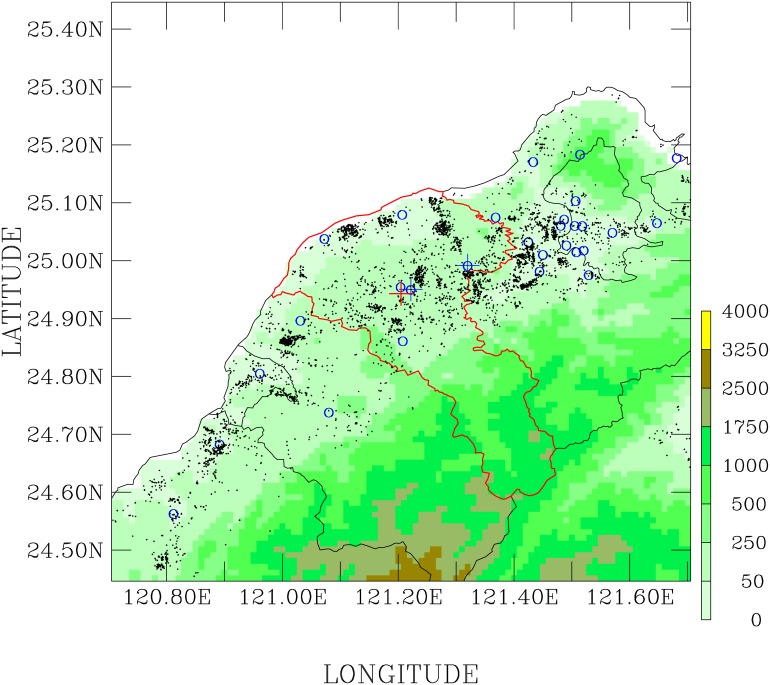
Spatial domain of Tao-Yuan City (marked by red colored curve), and terrain heights (shaded colors, in the unit of meters) distributed over northern Taiwan. Geographical locations of the Landseed Hospital (red corss); Chung-Li (a blue cross close to the red corss), Tao-Yuan (a blue cross on the right), and other ambient air monitoring stations (blue circles); and stationary air pollution sources (black crosses).

The medical records regarding the daily number of hospital outpatient visits for accidents in the Taiwan Landseed Hospital [121°12′18.36” *E*, 24°56′47.17” *N*; Fig 1] during the period of 2007–2011 were used in this work [6–9]. Outpatient data, selected with ICD-9 codes 800-949, were segregated in three age groups (0–15 years old, 16–65 years old, and greater than 66 years old) and two sexes (female and male).

Outpatient data from weekends (Saturdays and Sundays), national holidays (e.g., the Chinese New Year), and natural hazards (e.g., closure of public offices due to typhoons) were excluded from the analysis to eliminate artificial effects of zero outpatient visits for accidents during these periods. Because hospital emergency rooms are open continuously, hospital visits for accidents outside the normal working hours are admitted through emergency rooms. Consequently, we also conducted regression model analyses of all emergency visits for accidents.

### Ambient Air Pollution and Meteorological Data

Ambient air pollution and meteorological data were obtained from the Chung-Li ambient air pollution monitoring site (121°13′18.92” *E*, 24°57′15.17” *N*; Fig 1), which is approximately 2 km from Landseed Hospital. The Chung-Li ambient air monitoring site is located in the center of Tao-Yuan City, which contains a population of 2,096,432 people (as of September 2015), an area size 1,220 *km*^2^, and a population density of 1717 people *km*^−2^. (http://www/tycg.gov.tw/eng/). The highest population densities are in the 4 districts (Tao-Yuan, 416,403 people, 11,965 people *km*^−2^; Chung-Li, 380,829 people, 4978 people *km*^−2^; Ping-Jen, 212,073 people, 4441 people *km*^−2^; Bade, 135,885 people, 4031 people *km*^−2^) located between the Tao-Yuan and Chung-Li ambient air monitoring stations (Fig 1). These 4 districts contain more than 1 million people (1,145,190), and account for 55% of entire population in Tao-Yuan City. Most people live in areas with flat terrain, which are also characterized by high densities of industrial emissions (Fig 1). Hence, except for exceptional industrial emissions (such as industrial volatile organic compounds, hydrogen sulfide, etc), monitoring data from Chung-Li station is representative of Tao-Yuan City. For example, the *R*^2^ values for temperatures between Chung-Li and Tao-Yuan stations are 0.95 (2007), 0.98 (2008), 0.98 (2009), 0.98 (2009), and 0.99 (2011).

The hourly ambient data included the concentrations of 7 air pollutants and 5 meteorological parameters. The 7 air pollutants concentrations included those of particulate matter with particle diameters of less than 10 *μm* (*PM*_10_), particulate matter with particle diameters of less than 2.5 *μm* (*PM*_2.5_), ozone (*O*_3_), carbon monoxide (CO), nitrogen oxide (NO), nitrogen dioxide (*NO*_2_), and sulfur dioxide (*SO*_2_). The 5 meteorological parameters were temperature, precipitation, relative humidity, wind speed, and wind direction [10].

In a manner similar to the analysis of daily outpatient visits, data from the weekend and national holidays were excluded. This ensures that the time-series data regarding outpatient data, air pollution, and meteorological data covered consistent time periods. For the regression model analysis of hospital emergency visits, no public holidays were excluded because emergency rooms were open at all times. All data were analyzed for the period of 2007–2011. Air pollution and meteorological data used in this work can be openly accessed on the website of the Envrionmental Protection Administrtion (Taiwan) at http://www.epa.gov.tw.

### Test of Normal Distribution for Data

#### Data Distribution

The time-series data regarding hospital outpatient visits for accidents, air pollutants, and meteorological parameters were tested for normality. The procedures used to test the data regarding air pollutants, meteorological factors, and outpatient visits for normality are similar [10]. Test of normality for air pollution and temperature data were presented in a previous work [10]. The following are the steps that were used to test the data regarding outpatient visits for accidents for normality. The daily outpatient visit data for each age group were binned into 15 bins. For the outpatients with ages of 0–15 years and greater than 66 years old, the bin interval was 3, and the bin ranges were from 0 to 45 visits; for the 16–65 years-old outpatients, the bin interval was 12, and the bin ranges were from 0 to 180 visits. We then computed histograms of days (y-axis) versus daily outpatient visits binned into these 15 bins (x-axis). With these histograms determined, we computed standard deviations and mean values for each age group in each year of the period of 2007–2011.

#### Theoretical Normal Distribution

The mean value and standard deviation for an age group of outpatients in a year determined from the above method was then used to compute the theoretical normal distribution for the selected 15 bin intervals.

#### Test of Data Normality

Finally, with the data distribution and theoretical normal distribution determined, we could test the data for normality using a method such as the chi-squared test [11]. We used the chi-squared test for determining the goodness of fit to determine whether there was any difference between the observed (real-world) data and the expected (theoretical) data. The binned observed data were from the medical, meteorological, and air pollution data. The expected (theoretical) data were computed using the theoretical normal distribution with the mean and standard values determined from observations [11].

### Linear Regression Model Analysis

The processed time-series data regarding the daily outpatient visits were combined with each of the air pollutant-related and meteorological parameters for univariate regression model analysis to calculate correlation coefficients *R* [11]. Because there were 5 years of data (2007–2001), and 3 age groups of outpatients for each year, a total of 15 univariate regression model calculations (3 age groups per year x 5 years of data) were conducted for each air pollution-related and meteorological parameter.

For each pair of the outpatient-air pollution time-series data, a total of 15 correlation coefficients *R* were determined via 15 univariate regression model calculations. For each pair of the outpatient-meteorology time-series data, a total of 15 correlation coefficients *R* were determined via another 15 univariate regression model calculations. A total of 7 air pollutants required 105 univariate regression model calculations; and a total of 5 meteorological factors required 75 univariate regression model calculations. Hence, a total of 180 univariate regression model calculations were performed, and 180 correlations coefficients *R* were produced. These correlation coefficients *R* provided a good quantitative measure for analysis and comparison. The significances of each correlation coefficient *R* were measured using a *P*-value, which was obtained using the chi-squared test [11].

The univariate regression model was written in the Fortran language [12]. The model calculations were performed on the computers running an open source Linux operating system, Fedora Core [13]. The univariate regression model can also be run on the Windows operation system using the open source software package Cygwin [14]. The Cygwin package is a marvelous software package that enables development of Linux code in a Windows environment on a notebook computer. A detailed description of the algorithms and example code that were used in the univariate regression model calculations presented in this work can be found in reference 11.

### Multivariable Model Analysis

Multivariable regression models, which were based on the principles described in reference [15], were developed [10] and used to model the variation in the number of outpatient visits with respect to air pollutants and meteorological parameters for each year of the time period of 2007–2011. There were 7 air pollutant concentrations and 5 meteorological parameters (temperature, precipitation, wind direction, wind speed, and relative humidity) considered in the multivariable model analysis. The 7 air pollutants were *PM*_10_, *PM*_2.5_, *O*_3_, CO, NO, *NO*_2_, and *SO*_2_.

Table 1 presents a list of the multivariable regression models used in this work. Given the input time-series data of daily outpatient visits, air pollution variables, and meteorological variables, one can determine a correlation coefficient *β_i_* of a variable *i* for each multivariable model presented in Table 1 using a linear algebra method [11]. Here *i* = 1 indicates air pollution variable *PM*_10_, *i* = 2 indicates air pollution variable *PM*_2.5_, and so forth. The first 7 variables are related to air pollution factors, and the rest of the 5 variables are related to meteorological factors.

**Table 1:** List of Multivariable Regression Models.

Model1	OP=	*β*_0_+*β*_1_(*PM*_10_)
Model2	OP=	*β*_0_+*β*_1_(*PM*_10_)+*β*_2_(*PM*_2.5_)
Model3	OP=	*β*_0_+*β*_1_(*PM*_10_)+*β*_2_(*PM*_2.5_)+*β*_3_(*O*_3_)
Model4	OP=	*β*_0_+*β*_1_(*PM*_10_)+*β*_2_(*PM*_2.5_)+*β*_3_(*O*_3_)+*β*_4_(*CO*)
Model5	OP=	*β*_0_+*β*_1_(*PM*_10_)+*β*_2_(*PM*_2.5_)+*β*_3_(*O*_3_)+*β*_4_(*CO*)+*β*_5_(*NO*)
Model6	OP=	*β*_0_+*β*_1_(*PM*_10_)+*β*_2_(*PM*_2.5_)+*β*_3_(*O*_3_)+*β*_4_(*CO*)+*β*_5_(*NO*)+*β*_6_(*NO*_2_)
Model7	OP=	*β*_0_+*β*_1_(*PM*_10_)+*β*_2_(*PM*_2.5_)+*β*_3_(*O*_3_)+*β*_4_(*CO*)+*β*_5_(*NO*)+*β*_6_(*NO*_2_)+*β*_7_(*SO*_2_)
Model8	OP=	*β*_0_+*β*_1_(*PM*_10_)+*β*_2_(*PM*_2.5_)+*β*_3_(*O*_3_)+*β*_4_(*CO*)+*β*_5_(*NO*)+*β*_6_(*NO*_2_)+*β*_7_(*SO*_2_)+*β*_8_(*TEMP*)
Model9	OP=	*β*_0_+*β*_1_(*PM*_10_)+*β*_2_(*PM*_2.5_)+*β*_3_(*O*_3_)+*β*_4_(*CO*)+*β*_5_(*NO*)+*β*_6_(*NO*_2_)+*β*_7_(*SO*_2_)+*β*_8_(*TEMP*)+*β*_9_(*RAIN*)
Model10	OP=	*β*_0_+*β*_1_(*PM*_10_)+*β*_2_(*PM*_2.5_)+*β*_3_(*O*_3_)+*β*_4_(*CO*)+*β*_5_(*NO*)+*β*_6_(*NO*_2_)+*β*_7_(*SO*_2_)+*β*_8_(*TEMP*)+*β*_9_(*RAIN*)+*β*_10_(*WD*)
Model11	OP=	*β*_0_+*β*_1_(*PM*_10_)+*β*_2_(*PM*_2.5_)+*β*_3_(*O*_3_)+*β*_4_(*CO*)+*β*_5_(*NO*)+*β*_6_(*NO*_2_)+*β*_7_(*SO*_2_)+*β*_8_(*TEMP*)+*β*_9_(*RAIN*)+*β*_10_(*WD*)+*β*_11_(*WS*)
Model12	OP=	*β*_0_+*β*_1_(*PM*_10_)+*β*_2_(*PM*_2.5_)+*β*_3_(*O*_3_)+*β*_4_(*CO*)+*β*_5_(*NO*)+*β*_6_(*NO*_2_)+*β*_7_(*SO*_2_)+*β*_8_(*TEMP*)+*β*_9_(*RAIN*)+*β*_10_(*WD*)+*β*_11_(*WS*)+*β*_12_(*RH*)

OP: outpatient visits for accidents; TEMP: temperature; RAIN: rainfall; WD: wind direction; WS: wind speed; RH: relative humidity.

As for the univariate regression model described previously, the multivariable regression models were written in the Fortran programming language on a Linux platform running the open source operating system Fedora Core and can also be run using a Windows platform running the open source Cygwin package on a notebook computer. The Fedora Core operating system, Fortran compiler, and Cygwin package can be freely downloaded from open sources on the Web. Reference [15] provides plenty of useful examples and data, which are ideal for testing the multivariable regression model code developed in Fortran language [10]. Once the accuracy of these codes is verified, these multivariable regression models will be powerful tools for big data analysis, which requires efficient handling of the heterogeneous data sets that can easily exceed the capacity of commercial software packages.

In this work, there were 3 age groups of outpatients for a period of 5 years and a list of 12 variables for each age group of outpatients in each year to be modeled. Hence, a total of 180 (3 age groups x 5 years x 12 variables) multivariable regression model calculations were conducted to produce the analysis results presented in this work. These results include the coefficients *β_i_* for each variable *i*, *F* values, *R*^2^, and *P*-values to measure the statistical significance of the multivariable regression model calculations.

## Results

### Outpatient Visits During 2007–2011

Table 2 presents a list of annual outpatient visits for accidents during 2007–2011. The frequency of visits varied between 32,189 and 34,770. These data consistently demonstrated that there were more male accident outpatients (ranging between 18,271 and 20,049) than female outpatients (ranging between 13,610 and 15,038) each year. The number of male accident outpatients was approximately 1.31 to 1.47 times the number for females. More than 90% of patients that visited Landseed Hospital were from Tao-Yuan County (now called Tao-Yuan City) [6–9]. The population in the Tao-Yuan County is approximately equally divided between males and females, i.e., the male-to-females ranged between 1.03 to 1.01 during the study [Table 3]. Even accounting for the fact that accident attendance rates depend also on the distance from an accident and emergency department [16], the gender distribution of outpatient visits for accidents differed significantly from the population distribution (*P* < 0.0001; One-way ANOVA test, http://in-silo.net/tools/statistics/anova).

**Table 2:** List of Annual Outpatient Visits For Accidents.

Year	Male	Female	All	$\frac{Male}{Female}$
2007	19,622	13,907	33,529	1.41
2008	20,049	13,610	33,659	1.47
2009	18,271	13,918	32,189	1.31
2010	19,778	14,441	34,219	1.37
2011	19,732	15,038	34,770	1.31

**Table 3:** List of Annual Population in Tao-Yuan County (Now Called Tao-Yuan City)^a^.

Year	Male	Female	All	$\frac{Male}{Female}$
2007	981,486	953,482	1,934,968	1.03
2008	991,492	967,194	1,958,686	1.03
2009	999,065	979,717	1,978,782	1.02
2010	1,009,274	992,786	2.002,060	1.02
2011	1,013,618	999,687	2,013,305	1.01

^a^ Data source: End of Year (2013), Table 1, Population by sex and 5 year age group for counties and cities, Department of Household Registration, Ministry of Interior, http://www.ris.gov.tw/en/web/ris3-english/end-of-year.

One factor that could possibly cause more males than females to have accidents is that males tend to engage in more risky behaviors and get hurt more often than females [17]. Alcohol abuse was reported as a primary cause of accidents [18], and males may exhibit more frequent alcohol abuse than females. Moreover, there are more male than female drivers in the high-traffic urban areas that are typical of Tao-Yuan City, and more curbside parking can increase the risk of injury of child pedestrians by motor vehicles [19]. A study regarding injuries and the risk of disability in teenagers and young adults in England, Scotland, and Wales also observed that more males (62%) than females (26%) reported at least one accident that resulted in hospital treatment since age 16 [20].

**Fig 2:**
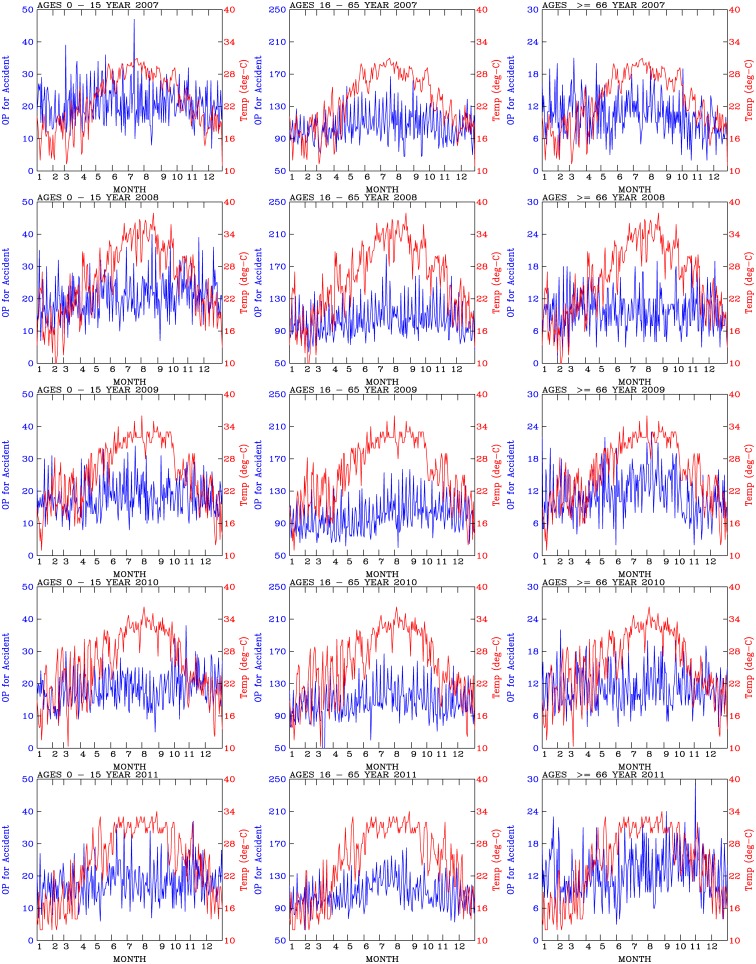
Time-series plots of the daily number of outpatients for accidents (blue curves; see the left y-axis for the scale) and temperatures (red curves; see the right y-axis for the scale).

Fig 2 shows time-series plots of daily temperatures and number of outpatients for accidents for each of the 3 age groups (0–15 years old, 16–65 years old, and greater than 66 years old) in each year during the period of 2007–2011. The seasonal trends in temperature, with low temperatures occurred during the winter season (December, January, and February), and high temperatures occurred during the summer season (June, July, and August), are readily identified from the plots. The temperatures varied between 12°*C* and 38°*C*. In addition to the pronounced seasonal variations in temperatures, day-to-day fluctuations in temperatures were also evident in the data.

In contrast with the sharp seasonal temperature variations, no distinctive seasonal variations in the number of outpatients for accidents were determined from the outpatient data. The number daily outpatient visits for accidents exhibited large day-to-day variations. Fewer accidents occurred in older people (greater than 65 years old) than the young people (0–15 years old and 16–65 years old). These results are consistent with the results of a previous work [21].

### Test of Normality for Outpatient Data

**Fig 3:**
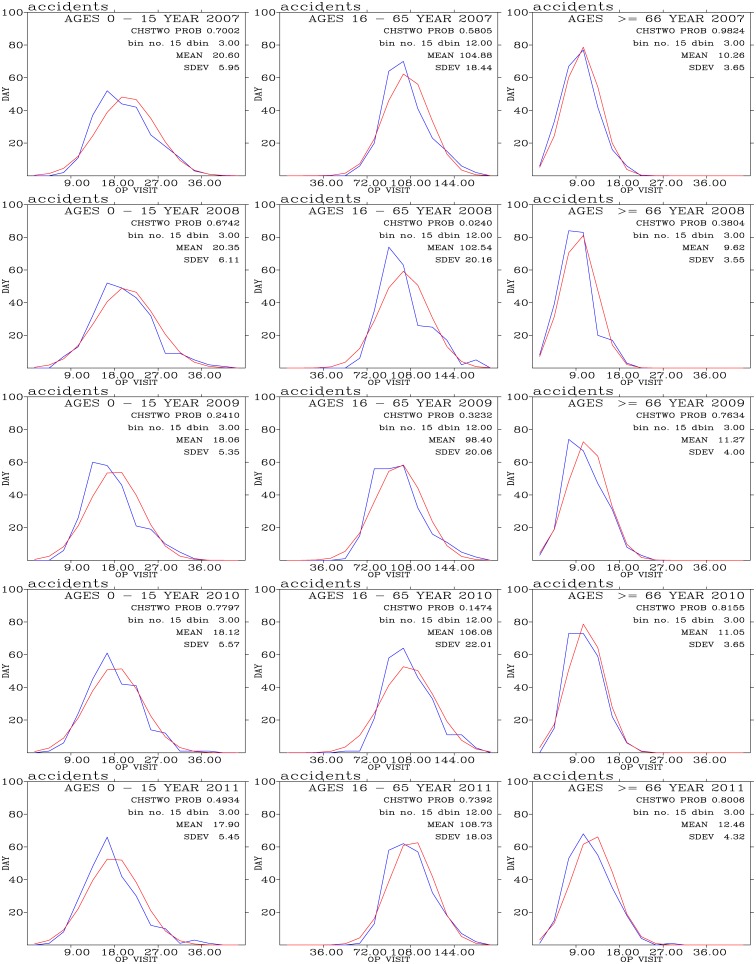
Normality test for the accident outpatient data. The frequency distribution of the daily number of outpatients is shown in blue, whereas the theoretical normal distribution is shown in red. The x-axis shows the ranges of the 15 bins (outpatients *day*^−1^). The y-axis shows the frequency of occurrence (days) for each of the 15 bins.

Fig 3 shows a normality test for the data regarding the number of outpatients for accidents in each of the 3 age groups during each year of the period of 2007–2011. Smaller values of *PROB* indicate that the outpatient data and the respective theoretical normal distribution were significantly different [11]. Our calculations yielded large vales of *PROB*, thereby indicating that outpatients for accidents were distributed nearly normally.

### Univariate Regression Model Analysis

**Fig 4:**
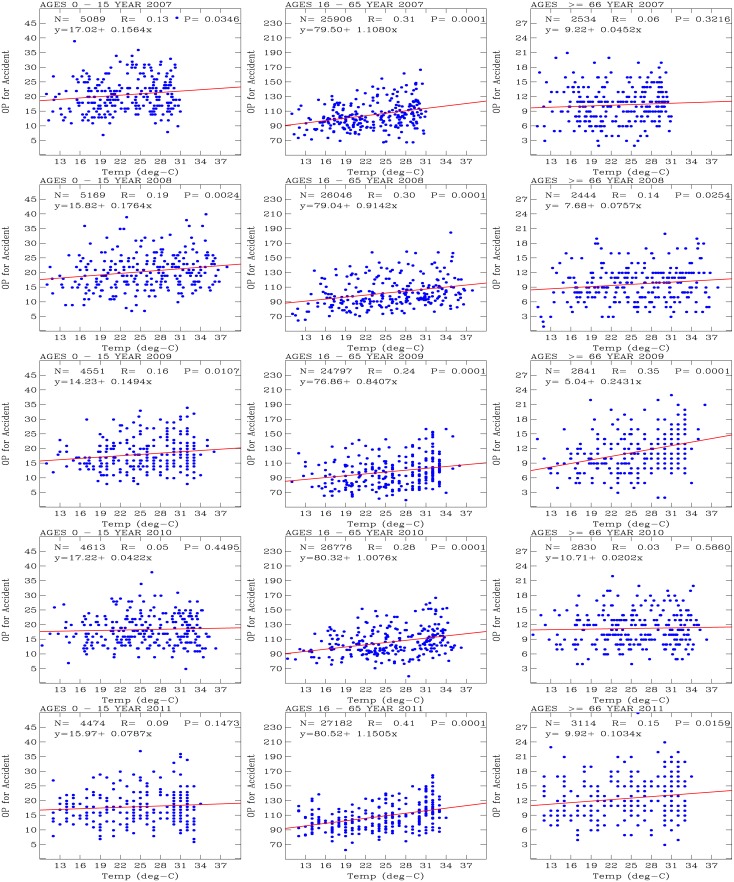
Scatter plots of daily number of outpatients for accidents (y-axis) versus temperature (x-axis). The 3 age groups of outpatients are shown in the left (0–15 years old), middle (16–65 years old), and right (greater than 66 years old) panels; the years are arranged from top (2007) to bottom (2011). Red lines show the results of a univariate regression performed on the data, with sample number *N*, correlation coefficient *R*, and measure of correlation significance *P*.

Fig 4 shows scatter plots of the daily number of outpatients for accidents (y-axis) versus temperature (x-axis). The 3 age groups of outpatients are shown in the left (0–15 years old), middle (16–65 years old), and right (greater than 66 years old) panels; the years are arranged from top (2007) to bottom (2011). Also shown on each plot is a red line, which indicates the results of a univariate regression performed on the data, with sample number *N*, correlation coefficient *R*, and measure of correlation significance *P*.

For the age group of 16–65 years old, the univariate regression models consistently indicated positive correlations for each year of 2007–2011, with correlation coefficients *R* that varied between 0.24 and 0.31. The coefficients for the *x* variables (i.e., temperature) of the linear equations varied between 0.8407 (in 2009) and 1.1505 (in 2011). These coefficients for *x* indicate that there was approximately one additional outpatient admitted for an accidents per one degree increase in temperature. The basic number of daily outpatients for accidents was approximately 76 to 80 outpatients per day when the temperature approached 0°*C* throughout these 5 years of analysis. This indicates a basic and stable characteristic of the statistics for the 16–65 years-old outpatients for accidents in the Tao-Yuan City area. These correlations are statistically significant (*P* = 0.0001).

The liner regression model analysis for the 0–15 years old outpatients also yielded positive correlation coefficients, with correlation coefficients *R* that varied between 0.05 and 0.19. The linear equations indicated that the basic number of outpatients per day was approximately 14 to 17 per day when the temperature approached 0°*C*. For the outpatients with ages of greater than 66 years old, the univariate regression models also indicated positive correlations. The linear equations indicated that the basic number of outpatients was 5 to 10 per day as the temperature approached 0°*C*, and the correlation coefficients *R* varied between 0.03 and 0.35. Statistically significant correlations (i.e., *P* < 0.1) occur when correlation coefficients *R* are large (i.e., *R* > 0.14). The larger the values for *R*, the smaller the *P*-values and the higher the significance of the statistical correlation and the univariate regression model analysis.

Statistically significant results (*P* < 0.1) for the 0–15 years old outpatients were obtained for 2007 and 2008, for which the *x* coefficients in the linear equations varied between 0.15 (2009) and 0.18 (2008). Similarly statistically significant correlations for the outpatients with ages of greater than 66 years old were obtained for 2008, 2009, and 2011. During these years, the *x* coefficients in the linear equations varied between 0.08 (2008) and 0.24 (2009).

These *x* coefficients in the linear equations indicated that the older people (greater than 66 years old) had the fewest accident per 1 degree increase in temperature, followed by the young people (0–15 years old). The middle-aged people (16–65 years old) were the group of outpatients that were most prone to accidents, with an increase of 1 accident per degree increase in temperature.

**Fig 5:**
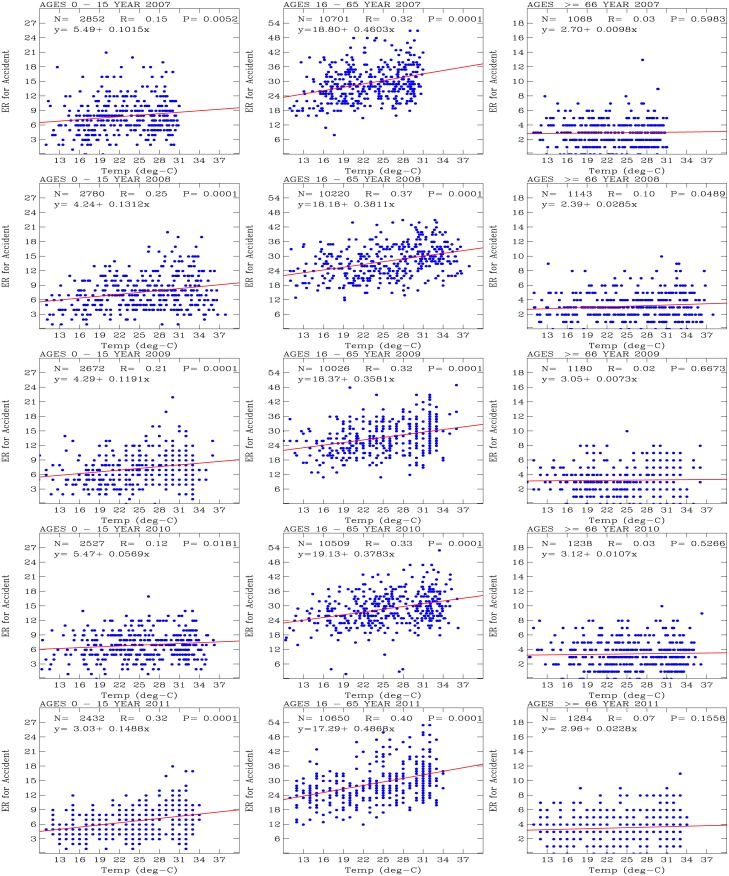
Scatter plots of number of daily emergency visits for accidents (y-axis) versus temperature (x-axis). The 3 age groups of outpatients are shown in the left (0–15 years old), middle (16–65 years old), and right (greater than 66 years old) panels; the years are arranged from top (2007) to bottom (2011). Red lines indicate the results of univariate regression performed on the data, with sample number *N*, correlation coefficient *R*, and measure of correlation significance *P*.

Fig 5 presents the regression model analyses of the number of hospital emergency room visits for accidents versus temperature during the study period for the three age groups of patients. Positive correlations between the numbers of emergency room visits and temperature were consistently obtained. These results are consistent with the positive correlations between the number of outpatients for accidents and temperature (Fig 4). We note that the coefficients *R* for the correlations between the number of emergency room visits and temperature were typically greater than the coefficients for outpatient visits for the 0–15 and 16–65 years-old patient age groups. However, for the patients aged greater than 66 years old, the correlation coefficients *R* for outpatients were greater than the coefficients for emergency room visits. Because hospital emergency rooms are open continuously, the consistently greater correlation coefficients *R* between the number of hospital visits for accidents and temperature compared with outpatient visits indicate significant correlations for the 0–15 and 16–65 years-old patient age groups.

In order to access time-lag effects on the correlation results shown here, Table 4 compares the correlation coefficients *R* for the daily hospital emergency room visits that are 0-day, 1-day, and 2-day lagging the daily temperature. For all the 45 scenarios analyzed (3 aged groups of outpatients X 5 years of data X 3 scenarios (0-day, 1-day, and 2-day delays)), 91% of scenarios show smaller correlation coefficients *R* for the 1-day and 2-day delay when compared with the no delay scenarios. Only 9% of the scenarios show equal correlations coefficients *R* when either the 1-day or 2-day delay are compared with the 0-day delay (no delay) scenarios. No scenario is found to have higher *R* from the 1-day and the 2-day delayed visits compared with the no delay visit.

**Table 4:** List of Correlation Coefficient *R* From the Univariate Regression Models for the Hospital Emergency Room Visits That are 0-day, 1-day, and 2-day Lagging to the Temperature Variables.

	0–15 Year Old			16–65 Years Old			>66*YearsOld*		
year	0-day	1-day	2-day	0-day	1-day	2-day	0-day	1-day	2-day
2007	0.15	0.10	0.10	0.32	0.31	0.30	0.03	<0.01	0.02
2008	0.25	0.21	0.20	0.37	0.37	0.33	0.10	0.07	0.06
2009	0.21	0.20	0.19	0.32	0.32	0.31	0.02	0.01	0.01
2010	0.12	0.05	0.05	0.33	0.31	0.30	0.03	0.01	0.03
2011	0.32	0.27	0.26	0.40	0.39	0.37	0.07	0.04	0.02

These results indicate the immediate effect of temperatures on the hospital emergency room visits. Interestingly, similar *R* occurs between the 1-day delay and 0-day delays for the 16–65 years old outpatients at some years (2008, 2009), but not for the young (0–15 years old) and the old (>65 years old) outpatients. This could reflect that, when the needs to immediately visit hospital emergency rooms occur, a more delayed character for the 16–65 years old outpatients compared with other two groups of outpatients.

### Comparing Outpatient Visits with Other Air Pollution and Meteorological Factors

How important is the temperature in determining the number of outpatient visits for accidents compared with air pollution and other meteorological factors? We used univariate regression models to compute correlation coefficients *R* and the significances of the correlations (*P*-values) for each of the 7 air pollutant concentrations and 5 meteorological factors listed above.

**Fig 6:**
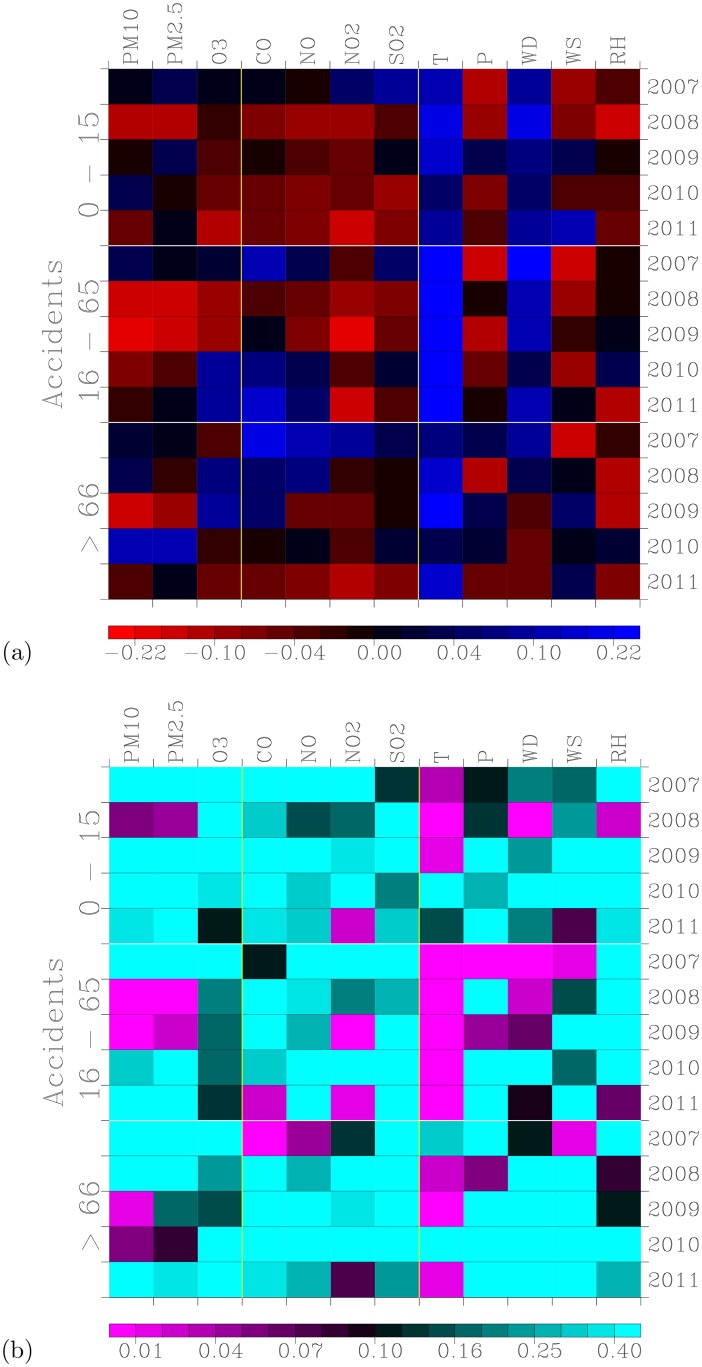
Correlation coefficients and measures of significance of correlations from the univariate regression model analyses for 3 age groups during the period of 2007–2011 (y-axis) versus air pollution and meteorological factors (x-axis). (a) Correlation coefficients *R* between number of outpatients for accidents and each of the air pollution and meteorological factors; (b) *P*-values of the significance of the correlations shown in (a).

Fig 6a shows the two-dimensional (2D) distribution of the correlation coefficients *R* calculated from the univariate regression model for the 3 age groups of patients during the period of 2007–2011.

This figure clearly shows that temperature was the most distinctive and persistent factor that determined the number of outpatient visits for accidents compared with other air pollution and meteorological factors. For the 16–65 years-old outpatients, the correlation coefficients *R* were consistently positive, with *R* values greater than 0.22 throughout the entire period of 2007–2011. The *P*-values for this group of outpatients during this 5-year period were very small, less than 0.01 (Fig 6b), thereby indicating statistically significant correlations.

For the other groups of outpatients, the coefficients for the correlations with temperature were also consistently positive. The larger the correlation coefficients *R*, the smaller the *P*-value (*P* less than 0.10), thus indicating the significance of the correlation.

For 12 of the parameters considered, Fig 6 reveals that only the temperature exhibited a persistent and significant correlation with the number of daily hospital outpatient visits for accidents throughout the 5-year analysis period. This is a key result of this work.

### Multivariable Regression Model Analysis: Significance of Temperature Factor

To further examine whether there are other potential confounding factors that could affect the correlation between temperatures and daily outpatient visits for accidents, we conducted a multivariable analysis for the 7 air pollution-related variables and 5 meteorological variables considered in this work.

Table 5 presents a list of coefficients *β*, *F* values, *R*^2^, and *P* values for the 12 multivariable regression models listed in Table 1. For multivariable regression models 1 through 7, the statistical significances of the model analyses were low, as indicated by the large *P* values. In model 8, when the temperature effect was considered in the model, the *P*-value decreased to 0.001, thus indicating statistical significance of the regression model, and *R*^2^ was equal to 0.11. Further inclusion of other meteorological factors, as demonstrated by models 9 through 12, slightly increased the *R*^2^ values to 0.15 while maintaining statistical significance, i.e., *P* values of less than 0.001.

**Table 5:** Coefficients and Statistical Values (*F*, *R*^2^, and *P*) for the Multivariable Regression Models for the 16–65 Years Old Outpatients for Accidents in 2007.

Model	*β*_1_(*PM*_10_) *β*_7_(*SO*_2_)	*β*_2_(*PM*_2.5_) *β*_8_(*TEMP*)	*β*_3_(*O*_3_) *β*_9_(*RAIN*)	*β*_4_(*CO*) *β*_10_(*WD*)	*β*_5_(*NO*) *β*_11_(*WS*)	*β*_6_(*NO*_2_) *β*_12_(*RH*)	*F*	*R*^2^	*P*
1	0.016								
							0.33	<0.01	0.564
2	0.041	-0.042							
							0.38	<0.01	0.685
3	0.040	-0.043	0.008						
							0.26	<0.01	0.857
4	0.031	-0.065	-0.006	1.848					
							0.93	0.02	0.448
5	0.019	-0.030	-0.039	4.687	-0.095				
							1.24	0.03	0.290
6	0.030	-0.011	-0.021	4.836	-0.076	-0.182			
							1.41	0.03	0.212
7	0.031	-0.021	-0.023	4.682	-0.074	-0.188			
	0.113						1.24	0.03	0.284
8	0.016	0.058	-0.115		-0.037	0.062			
	-0.011	1.333					3.63	0.11	0.001
9	0.005	0.050	-0.133	-0.370	-0.030	0.131			
	-0.093	1.368	-0.155				4.08	0.13	<0.001
10	0.003	0.043	-0.131	-0.327	-0.036	0.126			
	-0.067	1.171	-0.163	0.394			3.79	0.14	<0.001
11	0.013	0.030	-0.133	-0.870	-0.032	0.053			
	-0.097	1.122	-0.157	0.287	-3.241		3.75	0.15	<0.001
12	0.014	0.028	-0.124	-0.870	-0.031	0.051			
	-0.089	1.112	-0.165	0.286	-3.132	0.049	3.43	0.15	<0.001

Based on the changes of *R*^2^ and *P* values, the multivariable regression models revealed that the temperature variable was the dominant factor that associated air pollution-related and meteorological variables with the daily number of outpatient visits for accidents.

The multivariable regression models for 2008 (Table 6) indicated a significant change in *R*^2^ values from 0.04 to 0.16, and the *P* value decreased from 0.161 to less than 0.001 when the temperature factor was included in the model; for 2009 (Table 7), the *R*^2^ value changed from 0.08 to 0.10, and the *P* value decreased from 0.005 to 0.001; for 2010 (Table 8), the *R*^2^ value increased from 0.02 to 0.09, and the *P* value decreased from 0.600 to 0.003 when the temperature was included in the model.

**Table 6:** Coefficients and Statistical Values (*F*, *R*^2^, and *P*) for the Multivariable Regression Models for the 16–65 Years Old Outpatients for Accidents in 2008.

Model	*β*_1_(*PM*_10_) *β*_7_(*SO*_2_)	*β*_2_(*PM*_2.5_) *β*_8_(*TEMP*)	*β*_3_(*O*_3_) *β*_9_(*RAIN*)	*β*_4_(*CO*) *β*_10_(*WD*)	*β*_5_(*NO*) *β*_11_(*WS*)	*β*_6_(*NO*_2_) *β*_12_(*RH*)	*F*	*R*^2^	*P*
1	-0.075								
							6.90	0.03	0.009
2	-0.018	-0.088							
							3.81	0.03	0.023
3	-0.016	-0.085	-0.020						
							2.56	0.03	0.056
4	-0.007	-0.123	-0.039	4.676					
							2.32	0.04	0.058
5	-0.002	-0.123	-0.048	7.346	-0.032				
							1.94	0.04	0.089
6	-0.010	-0.128	-0.060	6.898	-0.038	0.080			
							1.67	0.04	0.130
7	0.002	-0.146	-0.065	7.094	-0.039	0.125			
	-0.200						1.52	0.04	0.161
8	0.036	-0.131	-0.233	-8.733	-0.021	0.463			
	-0.404	1.386					5.93	0.16	<0.001
9	0.034	-0.133	-0.237	-8.808	-0.019	0.472			
	-0.414	1.382	-0.045				5.29	0.16	<0.001
10	0.035	-0.134	-0.236	-8.727	-0.020	0.470			
	-0.417	1.371	-0.044	0.033			4.74	0.16	<0.001
11	0.047	-0.141	-0.226	-10.415	-0.017	0.359			
	-0.427	1.347	0.011	-0.076	-3.358		4.63	0.17	<0.001
12	0.049	-0.141	-0.217	-11.005	-0.017	0.356			
	-0.417	1.376	-0.010	-0.068	-3.174	0.105	4.26	0.18	<0.001

**Table 7:** Coefficients and Statistical Values (*F*, *R*^2^, and *P*) for the Multivariable Regression Models for the 16–65 Years Old Outpatients for Accidents in 2009.

Model	*β*_1_(*PM*_10_) *β*_7_(*SO*_2_)	*β*_2_(*PM*_2.5_) *β*_8_(*TEMP*)	*β*_3_(*O*_3_)*β*_9_(*RAIN*)	*β*_4_(*CO*) *β*_10_(*WD*)	*β*_5_(*NO*) *β*_11_(*WS*)	*β*_6_(*NO*_2_) *β*_12_(*RH*)	*F*	*R*^2^	*P*
1	-0.099								
							10.34	0.04	0.001
2	-0.131	0.067							
							5.40	0.04	0.005
3	-0.132	0.080	-0.050						
							3.70	0.04	0.012
4	-0.127	0.037	-0.054	1.300					
							3.17	0.05	0.014
5	-0.123	0.060	-0.125	4.767	-0.126				
							3.65	0.07	0.003
6	-0.120	0.117	-0.076	4.638	-0.106	-0.253			
							3.49	0.08	0.002
7	-0.120	0.114	-0.073	4.514	-0.106	-0.278			
	0.165						3.02	0.08	0.005
8	-0.101	0.113	-0.162	2.259	-0.077	-0.103			
	0.122	0.697					3.48	0.10	0.001
9	-0.110	0.105	-0.181	2.130	-0.068	-0.083			
	0.056	0.671	-0.230				3.78	0.12	<0.001
10	-0.114	0.110	-0.171	2.107	-0.065	-0.089			
	0.058	0.612	-0.229	0.246			3.46	0.13	<0.001
11	-0.114	0.108	-0.172	2.168	-0.066	-0.081			
	0.065	0.615	-0.232	0.261	0.396		3.14	0.13	0.001
12	-0.105	0.102	-0.148	1.866	-0.061	-0.075			
	0.088	0.698	-0.253	0.315	0.683	0.106	2.93	0.13	0.001

**Table 8:** Coefficients and Statistical Values (*F*, *R*^2^, and *P*) for the Multivariable Regression Models for the 16–65 Years Old Outpatients for Accidents in 2010.

Model	*β*_1_(*PM*_10_) *β*_7_(*SO*_2_)	*β*_2_(*PM*_2.5_) *β*_8_(*TEMP*)	*β*_3_(*O*_3_) *β*_9_(*RAIN*)	*β*_4_(*CO*) *β*_10_(*WD*)	*β*_5_(*NO*) *β*_11_(*WS*)	*β*_6_(*NO*_2_) *β*_12_(*RH*)	*F*	*R*^2^	*P*
1	-0.027								
							0.93	<0.01	0.336
2	-0.035	0.025							
							0.50	<0.01	0.610
3	-0.035	0.010	0.115						
							1.03	0.01	0.379
4	-0.032	-0.028	0.112	1.244					
							1.17	0.02	0.325
5	-0.033	-0.014	0.100	2.510	-0.050				
							1.02	0.02	0.406
6	-0.033	-0.003	0.107	2.377	-0.042	-0.051			
							0.86	0.02	0.522
7	-0.033	-0.002	0.108	2.561	-0.053	-0.085			
	0.181						0.79	0.02	0.600
8	-0.008	0.058	-0.049	-2.304	0.021	0.157			
	0.061	1.336					3.00	0.09	0.003
9	-0.008	0.057	-0.050	-2.308	0.021	0.158			
	0.058	1.335	-0.007				2.66	0.09	0.006
10	-0.008	0.057	-0.051	-2.335	0.020	0.165			
	0.061	1.344	-0.010	-0.065			2.39	0.09	0.010
11	-0.009	0.069	-0.051	-2.467	0.015	0.106			
	0.098	1.321	0.001	-0.163	-2.512		2.24	0.09	0.013
12	-0.006	0.060	-0.036	-2.741	0.021	0.107			
	0.113	1.337	-0.028	-0.170	-2.346	0.169	2.11	0.10	0.017

For 2011 (Table 9), the *R*^2^ values increased from 0.13 to 0.19, whereas the *P* value remain less than 0.001 in model 8, i.e., when the temperature variable was included in the model. Possible confounding factors occurred when CO was included, i.e., in model 4 (*R*^2^ increased from 0.01 to 0.04, and *P* decreased from 0.382 to 0.067), and when *NO*_2_ was included, i.e., in model 6 (*R*^2^ increased from 0.05 to 0.13, and *P* decreased from 0.020 to less than 0.001) in the year 2011 (Table 9).

**Table 9:** Coefficients and Statistical Values (*F*, *R*^2^, and *P*) for the Multivariable Regression Models for the 16–65 Years Old Outpatients for Accidents in 2011.

Model	*β*_1_(*PM*_10_) *β*_7_(*SO*_2_)	*β*_2_(*PM*_2.5_) *β*_8_(*TEMP*)	*β*_3_(*O*_3_) *β*_9_(*RAIN*)	*β*_4_(*CO*) *β*_10_(*WD*)	*β*_5_(*NO*) *β*_11_(*WS*)	*β*_6_(*NO*_2_) *β*_12_(*RH*)	*F*	*R*^2^	*P*
1	-0.007	<0.001							
							0.05	<0.01	0.827
2	-0.007	<0.001							
							0.02	<0.01	0.976
3	-0.031	<0.001	0.120						
							1.02	0.01	0.382
4	-0.052	<0.001	0.112	2.230					
							2.23	0.04	0.067
5	-0.044	<0.001	0.057	6.090	-0.147				
							2.74	0.05	0.020
6	0.011	<0.001	0.145	6.666	-0.096	-0.565			
							5.93	0.13	<0.001
7	0.014	<0.001	0.148	6.654	-0.088	-0.551			
	-0.178						5.10	0.13	<0.001
8	0.033	<0.001	0.016	1.582	-0.021	-0.243			
	-0.356	1.025					7.00	0.19	<0.001
9	0.033	<0.001	0.016	1.575	-0.021	-0.244			
	-0.355	1.025	0.003				6.20	0.19	<0.001
10	0.033	<0.001	0.021	1.257	-0.010	-0.233			
	-0.356	1.097	0.003	-0.254			5.63	0.19	<0.001
11	0.035	<0.001	0.025	1.107	-0.010	-0.251			
	-0.371	1.101	0.002	-0.292	-0.913		5.11	0.19	<0.001
12	0.036	<0.001	0.002	1.098	-0.006	-0.242			
	-0.400	1.095	0.031	-0.335	-1.119	-0.147	4.75	0.19	<0.001

For these 5 years of analysis with the multivariable regression models, the *R*^2^ values varied between 0.10 and 0.19, with *P* values less than 0.017. These results indicated that the multivariable regression model analyses were statistically significant. The coefficients *β* for the temperature variable vary between 0.7 and 1.4, consistent with the results from the univariate regression models.

As similar to Table 4 for the univariate model analysis, we run the multivariable models through the 180 scenarios (1 aged group of outpatient X 5 years of data X 12 multivariable models X 3 scenarios (0-day, 1-day, and 2-day delays)) to investigate the sensitivity of the time-lag effects on the results shown above. Table 10 shows a list of *R*^2^ from 180 scenarios. Examinations of these results show no increases in *R*^2^ when the 1-day and 2-days delays in the hospital emergency room visits are taken into account. Also, the temperature variable (included in models 8 and after) remains to be the dominating factor in associating with the hospital emergency room visits.

**Table 10:** List of *R*^2^ From the Multivariable Regression Models for the Hospital Emergency Room Visits of the 16–65 Years Old Outpatients That are 0-day, 1-day, and 2-day Lagging to the Variables.

Year	2007			2008			2009		
Model	0-day	1-day	2-day	0-day	1-day	2-day	0-day	1-day	2-day
1	<0.01	0.01	<0.01	0.01	<0.01	0.01	<0.01	<0.01	0.01
2	0.01	0.02	0.01	0.01	0.01	0.01	<0.01	<0.01	0.01
3	0.01	0.02	0.03	0.03	0.02	0.03	0.01	0.03	0.02
4	0.01	0.03	0.04	0.04	0.07	0.07	0.01	0.04	0.06
5	0.01	0.03	0.04	0.05	0.07	0.07	0.05	0.05	0.06
6	0.05	0.05	0.06	0.09	0.09	0.08	0.09	0.06	0.07
7	0.06	0.05	0.06	0.09	0.09	0.09	0.09	0.06	0.07
8	0.15	0.11	0.11	0.16	0.15	0.12	0.15	0.11	0.12
9	0.15	0.11	0.11	0.17	0.15	0.13	0.15	0.11	0.12
10	0.15	0.11	0.11	0.17	0.15	0.13	0.16	0.11	0.12
11	0.15	0.11	0.12	0.18	0.15	0.13	0.16	0.11	0.12
12	0.16	0.12	0.13	0.18	0.16	0.14	0.16	0.11	0.12
Year	2010			2011					
Model	0-day	1-day	2-day	0-day	1-day	2-day			
1	0.01	0.01	0.01	0.01	<0.01	<0.01			
2	0.01	0.01	0.01	0.01	0.01	0.02			
3	0.01	0.01	0.02	0.01	0.01	0.02			
4	0.01	0.01	0.02	0.02	0.03	0.06			
5	0.02	0.02	0.04	0.02	0.03	0.07			
6	0.06	0.05	0.05	0.12	0.08	0.10			
7	0.06	0.05	0.07	0.12	0.08	0.11			
8	0.14	0.10	0.11	0.23	0.18	0.16			
9	0.14	0.10	0.11	0.23	0.21	0.17			
10	0.14	0.11	0.11	0.23	0.21	0.17			
11	0.14	0.11	0.12	0.23	0.21	0.17			
12	0.14	0.11	0.12	0.23	0.21	0.18			

Table 9 shows the occurrence of confounding factors CO and *NO*_2_ in 2011. In order to investigate the possible causes of these two confounding factors, we run multivariable models for temperature with respect to variables from air pollutants for the period 2007–2011. Table 11 shows results of *β* for each air pollutant when the following multivariable model is used: *T* = *β*_1_(*PM*_10_)+*β*_2_(*PM*_2.5_)+*β*_3_(*O*_3_)+*β*_4_(*CO*)+*β*_5_(*NO*)+*β*_6_(*NO*_2_)+*β*_7_(*SO*_2_). Table 12 shows evolution of *R*^2^ when each of the air pollutant variables is successively included in the multivariable models. The *β* coefficients (Table 11) show that temperature is positively associated with *O*_3_ and *CO*, negatively associated with NO and *NO*_2_, and the associations between temperature and other pollutants (*PM*_10_, *PM*_2.5_, and *SO*_2_) are weak.

**Table 11:** List of *β* From the Multivariable Regression Model for the Temperature With Respect to Air Pollutants: *T* = *β*_1_(*PM*_10_) + *β*_2_(*PM*_10_) + *β*_3_(*O*_3_) + *β*_4_(*CO*) + *β*_5_(*NO*) + *β*_6_(*NO*_2_) + *β*_7_(*SO*_2_).

Year	2007	2008	2009	2010	2011
*β*_1_(*PM*_10_)	0.01	-0.04	<-0.01	-0.01	-0.01
*β*_2_(*PM*_2.5_)	-0.05	-0.01	-0.03	<0.01	<0.01
*β*_3_(*O*_3_)	0.08	0.12	0.15	0.12	0.14
*β*_4_(*CO*)	3.70	11.80	3.62	2.21	4.93
*β*_5_(*NO*)	-0.02	-0.02	-0.05	-0.01	-0.05
*β*_6_(*NO*_2_)	-0.20	-0.20	-0.26	-0.28	-0.33
*β*_7_(*SO*_2_)	0.07	0.10	0.03	0.14	0.11

**Table 12:** Evolution of *R*^2^ From the Multivariable Regression Models *T* = *β*_1_(*PM*_10_) + *β*_2_(*PM*_10_) + *β*_3_(*O*_3_) + *β*_4_(*CO*) + *β*_5_(*NO*) + *β*_6_(*NO*_2_) + *β*_7_(*SO*_2_) When Each of Air Pollutant is Successively Included.

Year	2007	2008	2009	2010	2011
*PM*_10_	0.01	0.02	0.01	0.02	<0.01
*PM*_2.5_	0.04	0.02	0.01	0.02	0.01
*O*_3_	0.10	0.13	0.13	0.11	0.11
*CO*	0.28	0.31	0.23	0.19	0.28
*NO*	0.31	0.33	0.29	0.23	0.31
*NO*_2_	0.44	0.39	0.41	0.37	0.50
*SO*_2_	0.44	0.39	0.41	0.38	0.50

As demonstrated in Table 12, when the multivariable model for temperature contains only *PM*_10_ and *PM*_2.5_ two variables, the *R*^2^ values are low (less than 0.04) and statistically not significant. As *O*_3_ been included in the model, *R*^2^ values rise to 0.10 and 0.11, indicating that *O*_3_ levels are positively associated with temperature. This is consistent with higher temperatures often occur during the day-time and cloud-free conditions, resulting in atmospheric conditions that are conducive for photochemical *O*_3_ production.

The inclusion of CO variable further enhances *R*^2^ values to 0.19 to 0.31, indicating that high temperatures are associated with high CO levels. Since vehicular emissions are major sources of CO in urban areas in Taiwan [22], this positive association indicate enhanced vehicular emissions and vehicular usages as temperatures become higher.

The next significant increases in *R*^2^ values occur when *NO*_2_ variable is included in the model. As *NO*_2_ is also a main pollutant from vehicular emissions, high *R*^2^ values demonstrate again the importance of vehicular sources in the study area. The negative coefficient for *NO*_2_ variable, while positive coefficients for *O*_3_ and CO clearly indicate effective photochemical production of *O*_3_ from *NO*_2_ while emissions of CO are sustained in the urban environment.

Detailed causes of each accident are not available. However, the multivariable models predict that increase in vehicular usage and emissions are strongly associated with increase in temperatures, which in turn are strongly associated with increase in the number of hospital outpatient visits for accidents. Hence, it is likely that the causes of accidents are related to the increases in vehicular usages and emissions during the time periods with high temperatures. More vehicles and higher driving speeds can result in increase in CO and *NO*_2_ emissions, increase in *O*_3_ levels [23], more accidents, and more hospital usage. This analysis is consistent with the hospital clinical experience, which showed that most (about 60%–80%) of the patients for accidents were related to traffic accidents, followed by accidents occurred in work places. Table 12 shows exceptional high *R*^2^ values of 0.50 in for model when *NO*_2_ is included as a model variable compared with other years. As a result, *NO*_2_ variable also exhibits distinctive association with the hospital outpatients for accidents in 2011 than other years.

## Conclusions

An accident is an unwanted hazard for any person. However, accidents do occur. It is important to analyze accident data accumulated from people seeking medical care to understand the history of accidents that have occurred such that we can develop methods to reduce the risk of accidents in the future. In this work, we searched for correlations between daily accident rates and daily air pollution and meteorological factors. Based on the daily number of hospital outpatients admitted for accidents during a 5-year period, 2007–2011, data for a total of 168,366 outpatients were analyzed with univariate regression models and multivariable regression models.

Our analysis demonstrated that the number of male outpatients admitted for accidents was approximately 1.31 to 1.47 times the number of females; the difference was statistically significant because the population is approximately equally distributed between males and females (*P* < 0.0001). For all 12 (air pollution and meteorology) of the parameters considered, only the daily temperature exhibited consistent and significant correlations with the daily number of hospital outpatient visits for accidents throughout the 5-year analysis period.

The univariate regression models indicated that older people (greater than 66 years old) had fewer accidents per 1-degree increase in temperature, followed by young people (0–15 years old). Middle-aged people (16–65 years old) were the group of outpatients that were most prone to accidents, with an increase of 0.8–1.2 accidents per degree increase in temperature.

The multivariable regression models also revealed that the temperature variable was the dominant factor in associating air pollution and meteorological variables with the daily number of outpatient visits for accidents. The results of the multivariable regression model analyses were statistically significant. The accident rates of 0.7–1.4 additional accidents per 1-degree increase in temperature were consistent with the results of the univariate regression models. Our further multivariable model analysis of temperature with respect to air pollution variables show that, through the increases in emissions and concentrations of CO, photochemical O3 production and NO2 loss in the ambient air, increases in vehicular emissions are associated with increases in temperatures. As such, increases in hospital visits for accidents are related to vehicular emissions and usage. This finding is consistent with clinical experience which shows about 60% to 80% of accidents are related to traffic, followed by accidents occurred in work place.

The accident data analyzed in this work were selected according to the ICD-9 codes 800-949. Follow-up works to analyze the detailed nature of each accident as documented according to the ICD-9 codes to further explore how the nature of accidents on changes in meteorology and climate factors are ongoing.

## Supporting Information

S1 File Daily meteorology, air pollution, and hospital visit data used in this work. The daily meteorological data (temperature, relative humidity, wind direction, wind speed, rainfall), air pollution data (*PM*_10_, *PM*_2.5_, *O*_3_, CO, NO, *NO*_2_, *SO*_2_), and hospital visits for accidents (outpatient visits, emergency room visits) for each year of the 2007–2011 study period are included in the uploaded data file. There is also a README file, containing the detailed file structures for the data set, and the description for each data file. Interested readers can contact Kuo-Ying Wang at kuoying@mail.atm.ncu.edu.tw to access data as described above. Alternatively, interested readers can use following methods to obtain data (meteorology and air pollution) from Taiwan Environmental Protection Administration (EPA): 1) Click on http://taqm.epa.gov.tw/taqm/en/default.aspx; 2) Look on a list of items from the left column shown from the pop-up window. Search and click on an item called “Data Service”, which will lead to “Air Quality”. Then click on it to see “Hourly Value”. Then click on “Hourly Value” to download hourly data for all ambient air monitoring sites in Taiwan.
(TAR)
